# Only shine the light where and when it is needed – the impact of light pollution

**DOI:** 10.1016/j.fhj.2025.100470

**Published:** 2025-09-20

**Authors:** Derrick Wayne Thomas, Mario Motta, John Barentine

**Affiliations:** a University Hospitals Plymouth NHS Trust, Plymouth, Devon, PL6 8DH, United Kingdom; b Tufts University School of Medicine, Harrison Avenue, Boston, MA 02111, United States; c DarkSky Consultancy LLP, 9420 E Golf Links Rd, Tucson, AZ 85730, United States

## Abstract

There is growing evidence that artificial light at night (ALAN) has an adverse effect on human health and ecology. Long-term global studies also show that the world is getting brighter at night. This technological advancement comes at a price to both humans and nocturnal species that share our planet. Light pollution is wasted energy when the world is in simultaneous climate, biodiversity and energy security crises. There are no known negative consequences of reducing light pollution. A vital next step is to make ‘quick win’ changes by following the basic principles of using light only where it is needed, in the right amount, in the right place, at the right time and with the right colour. We advocate for more research into the adverse effects of ALAN on human health to help drive these changes and allow people to make informed choices about how light is used at local, national and international levels.

## Article

In 1950, Doll and Hill published the link between smoking and carcinoma of the lung.^1^ Fifty-seven years later England banned smoking in enclosed public spaces. This legislation recognised the potential harm of smoking on others who were exposed to this toxin beyond their own control. When it comes to similar concerns such as artificial light at night (ALAN), consisting of home, street and business lighting, the public often have no choice other than to accept this manmade development. Little is done to frame this as the public health issue it is: ALAN is a pollutant with adverse human and environmental effects.^2^^,^^3^ Humans require darkness at night to maintain a normal circadian rhythm. Darkness leads to melatonin release, an adjuvant to the immune system. The immune system’s effects against invading micro-organisms is well recognised, yet its link with suppressing and removing cancer cell development is underappreciated. We all make billions of new cells daily, and a very small number produced are abnormal. Most abnormal cells are non-viable, but a few develop into cancer cells. Suppression of melatonin at night diminishes adjuvant immune activity of B- and T-cell activity. This increases the chances that abnormal cancer cells naturally produced can escape detection and proliferate.^4^ A very low light level of 5 lux (∼5 candles) is enough to suppress melatonin release, even while asleep.^5^

The intensity of light is not the only issue, but rather modern light-emitting diodes (LEDs) emit a much broader spectrum which includes the blue, more harmful part of the spectrum, compared to firelight for example. A balance between sleep and awake states has developed in animal species over millennia, and we disrupt such things at our peril. Furthermore, ALAN has pronounced adverse biological effects on plants and animals, both through a direct physiological impact and through its interactions with other species in the same environment. Examples include birds being drawn away from their migratory routes by bright city lighting, increasing their risk of collision with high-rise buildings and exposure to city air pollution. A significant proportion of insect species are active at night and disruption of their normal lighting conditions has been shown to lead to harm in nocturnal pollinator species that in turn could reduce crop yields and affect agricultural food supplies.^6^ From a purely anthropocentric view alone, this is concerning.

Considering the impact of ALAN on the human immune system, we observe the association between satellite-indicated ALAN and its correlation with the geographic distribution of cancer. Our knowledge is incomplete, and we currently lack strong evidence for causal connections. Each year more evidence is published of the adverse effects of exposure to ALAN and its association with sleep disorders, macular degeneration, depression, obesity, heart disease, diabetes and cancers, and more,^6^ of which breast and prostate cancer appear to show the strongest correlations.^7^

One can be critical of drawing conclusions by correlating low-resolution satellite data with the spatial distribution of patients with diseases. It does not fully account for exposure to light in the home or other environmental influences, or how much of a lighting dose people are getting at ground level in proportion to what satellites detect. We therefore rely on epidemiological data correlated with the satellite-detected light intensity. In 2017 the Nursess Health Study II looked at a population of over 130,000 and adjusted for the known important risk factors for breast cancer, showing that there was still a 14% increase in breast cancer risk where ALAN was highest.^8^ Since then a number of other studies have confirmed these results, including in 2018 a large study from Spain and the EU.^7^ In 2012 the American Medical Association (AMA) published a paper documenting the adverse medical effects of light pollution on humans and the environment, and in 2016 this formed the basis of the AMA’s recommendation that all street lighting should use light sources with correlated colour temperatures of 3,000 K or less.^9^^,^^10^ Researchers have only recently begun to directly measure light exposures with wearable sensors. One large cohort study using this approach found a statistically significant increase in all-cause mortality among participants who had high night-time light exposures.^11^ The result persisted after accounting for demographic and lifestyle influences.

People make choices about their own health based on sound evidence, and they need to feel that they have the agency to understand and control relevant factors when they are known or suspected to be hazardous. The impact of ALAN is no different, and the solutions are straightforward: use light only where and when it is needed, in the right amount, in the right place, at the right time, and of the right colour. This applies equally to lighting in outdoor and indoor spaces, as the expected human biological effects of ALAN result from the sum of exposures in both spaces. In the indoor space, aim to limit night-time exposure to short wavelengths of artificial lighting by the use of warmer-coloured (<3,000 K) lamps. This is particularly important before going to bed. In the outdoor space, use lighting again in the warmer part of the spectrum; only when needed; directed to where needed; and shielded. Upon seeing bright lighting, ask: ‘what is its purpose?’ and ‘is the nature of that lighting meeting or exceeding that purpose?’.

Research shows no clear connection between outdoor lighting and either crime or traffic safety.^12^ Yet fear of crime, and safety considerations, appear to be behind much of the proliferation of lighting at night. Because LED-based lighting costs are low, the availability of unsuitable lighting products has increased. The money saved from replacing older lighting technologies with highly energy-efficient LEDs may have fuelled the observed global proliferation of poor-quality lighting, preventing any net environmental benefit.^13^ The end result is that the world at night is getting brighter at a global average rate of some 10% per year,^2^ even as it is becoming no safer for it.

In 2023, a House of Lords Science and Technology Committee enquiry found that ALAN ‘contributes to a range of adverse health outcomes’, and that it is ‘poorly understood and poorly regulated’. It advised that there should be more research into the effects of light pollution.^14^ However, the UK government at the time rejected the recommendation for a research programme of studies.^15^ We feel that deepening our understanding of ALAN is a vital next step. Without it, we will also struggle to help citizens who are concerned about the increasing levels of ALAN and its environmental impact to make informed decisions. If we include the known adverse effects of ALAN on other planetary species and the climate, as well as the financial impact of in the form of energy waste, surely it makes sense to only use ALAN where evidence shows it can have a positive impact. Just like passive exposure to smoking, will we have to wait another 50 years before we recognise that we could and *should* have done something about this earlier?

## Declaration of competing interest

The authors declare the following financial interests/personal relationships which may be considered as potential competing interests:

JB (John Barentine, co-author) is the Principal Consultant at Dark Sky Consulting, LLC. He is a PhD professional astronomer. He has worked in public policy, media and education related to the adverse effects of light at night. He has created numerous policy documents around night-time lighting and has published extensively on light pollution and public policy. If there are other authors, they declare that they have no known competing financial interests or personal relationships that could have appeared to influence the work reported in this paper.
